# Mental health services in Cambodia: an overview

**DOI:** 10.1192/bji.2019.24

**Published:** 2020-05

**Authors:** Sarah J. Parry, Ewan Wilkinson

**Affiliations:** 1 MBBS, BSc (Hons), Researcher, OMF International, Phnom Penh, Cambodia. Email: sarah316103@gmail.com; 2 MBChB, FFPHM, Visiting Professor of Global Public Health, Institute of Medicine, University of Chester, UK

**Keywords:** Low- and middle-income countries, rebuilding, strategic plan, mental health service development, Cambodia

## Abstract

Mental health services in Cambodia required rebuilding in their entirety after their destruction during conflict in the 1970s. During the late 1990s there was rapid growth and development of professional mental health training and education. Currently, basic mental healthcare is available primarily in urban areas and is provided by a mixture of government, non-government and private services. Despite the initial rapid growth of services and the development of a national mental health strategy in 2010, significant challenges remain in achieving an acceptable, standardised level of mental healthcare nationally.

Cambodia is a country in Southeast Asia, bordered by Thailand, Laos and Vietnam, with a population of 15.8 million.^1^ The population remains predominantly rural although the distribution reduced from 81% to 68% rural between 1998 and 2016.^1^ The national poverty rate declined from 48% in 2007 to 13% in 2014.^2^

The majority (97%) of the population is Buddhist.^3^ The Cambodian concept of health is complex, rooted in Hindu-Buddhist beliefs, animistic spiritual beliefs, the concept of luck and astrology, and physical/somatic concepts.^4,5^ Religious and traditional healers play an important role in physical and mental healthcare in Cambodia.^3,4^ Traditional approaches to healthcare are usually used first, particularly in rural areas.^4,6,7^ As in many cultures, there remains significant stigma associated with mental disorders in Cambodia.^6,8^

The people of Cambodia experienced a prolonged period of intense conflict, loss and societal disruption between 1967 and 1975 owing to civil war, followed by the Khmer Rouge period until 1979, with up to one-third of the population dying from starvation, disease or execution. In 1975, Cambodia had just two psychiatrists, running a single 800-bed psychiatric hospital with a patient population of approximately 2000.^5,9^ During the Khmer Rouge period the mental health services in their entirety were destroyed, leaving no psychiatrists or other trained mental health professionals.^5,6,9,10^ When the Paris Peace Agreement was signed in 1991, marking the official end of the Cambodian–Vietnamese War Cambodia had to completely rebuild the health, education and community services.^4,5,7^ The long-lasting effects of genocide and war compound the challenge of rebuilding the country, as complex trauma continues to affect the mental well-being of the people of Cambodia.^3,7^ Available epidemiological data show a high prevalence of substance misuse, neurological and mental disorders compared with normative populations.^4,7,8^

## Rebuilding mental health services in Cambodia

### Training

In 1992, the Mental Health Subcommittee of the Cambodian Ministry of Health was formed to develop the country's mental health services.^9^ Between 1994 and 2004, partnerships with the International Organization for Migration and the University of Oslo trained 26 psychiatrists^5,7,9,10^ and 40–45 psychiatric nurses.^6,9–11^ A combined total of 600 nurses and primary care doctors were trained in basic mental healthcare^6,10,11^ through partnerships including the Harvard Training Program.^5,7,11^

In 2005, the Cambodian University of Health Sciences took over the 3-year psychiatry residency training programme.^3,10^ There are currently approximately 60 psychiatrists in Cambodia: 1 per 260 000 people,^3^ compared with 7068 (1 per 9300 people) in the UK.^12^

Despite the rapid early growth in the number of mental health professionals, when international funding ended the training opportunities reduced.^3,5,7^ There has been no psychiatric nurse training in the country since 2006.^5,10^ Furthermore, it was estimated in 2012 that 30% of the psychiatrists and 90% of the primary care physicians who had received the basic mental health training were no longer involved with clinical mental healthcare.^4,11^ Many professionals have left to work in other medical specialties^4,10,11^ or for non-governmental organisations (NGOs).^10^

The Royal University of Phnom Penh has offered a bachelor's degree in psychology since 1994 and a master's degree in clinical psychology and counselling since 2008.^4^ The university's Department of Social Work, established in 2008, offers both bachelor's and master's programmes in social work.^4^ However, there are no established posts for psychologists or social workers in public hospitals^3^ and most graduates work for NGOs.^8^

### Government services

In 2012, the total health expenditure in Cambodia was US$1033 million.^2^ Government spending comprised just under 20% of this figure: US$199.1 million. Of the total government health spending, it was estimated that 0.02% was on mental health.^6^

There are 25 provinces in Cambodia, including the capital city Phnom Penh. In 2015, there were 1141 community health centres, 102 referral hospitals, 25 provincial referral hospitals and 9 national hospitals in Phnom Penh. There is no current systematic referral pathway between health centres and hospitals, which is a significant barrier for delivering integrated mental healthcare.^3^

The rapid growth in mental health services through international funding in the late 1990s led to government mental health clinics operating in 95% of provinces and three in-patient psychiatry units were available for emergency assessments by 2007.^9^ However, in 2010 this had reduced to two in-patient psychiatry units providing a total of 14 beds,^11^ and 60% of the referral hospitals and 2% of community health centres provided mental health services.^11^ The number of government in-patient beds has remained low, at 10–15, since 2010.^3–6,11^ In 2018, the few specialist out-patient mental health services were predominantly located in urban centres.^3^

The Centre for Child and Adolescent Mental Health is the only specialist child and adolescent mental health service in Cambodia; it is a government service supported by an international NGO.^3,11^

In 2012, there were between 11 and 14 drug treatment and social affair centres in Cambodia.^6^ These centres were designed to address drug dependence and provide social rehabilitation services. A community-focused approach to addressing the high prevalence of substance misuse in Cambodia has since been proposed.^7,11^

### Non-governmental services

A significant proportion of healthcare in Cambodia is provided by private for-profit and private not-for-profit services, which operate alongside the government health services.^2^ In 2015, there were 8488 registered private healthcare facilities and over 180 healthcare-related NGOs in Cambodia.^2^ A few of these organisations provide mental healthcare services. People with mental disorders may be restrained at home as relatives are not aware of other options; this is a focus for some human rights organisations.^4,6^ NGOs tend to work independently and access to the services they provide depends on geographical location and target population.^6^

Psychosocial services frequently focus only on meeting immediate basic needs, including shelter and protection,^8^ and there are limited services providing psychological therapies.^6^ When services are accessed, there is often a reliance on medication alone rather than addressing psychological needs, owing to the lack of resources.^6^ However, some NGOs are currently focusing on providing psychological services, including trauma-focused therapy and eye-movement desensitisation therapy.^8^ A number of psychological services are also available through the rapidly growing private health sector^2,4,6^ and a small number of private in-patient psychiatric beds are available.^4,10^

### Availability of medication

There is currently limited availability of psychotropic medications, particularly in rural areas.^3^ There is no unified regulation of how or by whom psychotropic drugs are prescribed.^3^ Patients are frequently prescribed several psychotropic medications without being given information on the drugs or why they have been prescribed.^4,6^ In 2012, the psychotropic medication available was mainly older generations of pharmaceuticals^4,6^ and medication shortages were frequent.^6^

## Development of a mental health strategy

In 2010, the Cambodian government issued the Mental Health and Substance Misuse Strategic Plan for 2011–2015.^11^ This outlined the vision, mission and strategy for development of the mental health services in Cambodia as well as some of the key challenges faced. The vision was for ‘All Cambodian people [to have a] high level of mental health and psychosocial well-being, contributing to the quality of life’ and the mission was ‘To ensure all Cambodian people will have access to the highest quality mental health and substance abuse services’.^11^

Despite this promising strategic plan there have been several challenges to its implementation and limited progress has been made since the initial rapid growth in the late 1990s. It was acknowledged in the subsequent Health Strategic Plan for 2016–2020 that the system was ill-equipped and provided limited services.^2^ Mental healthcare funding remains low, human resources remain limited^3^ and training initiatives and projects frequently rely on external funding.^7,11^ Developing legislation and regulation for delivering mental healthcare was identified as a goal in the strategic plan,^11^ but there are currently no national clinical guidelines for diagnosis and treatment of mental disorders and no mental health legislation in Cambodia.^3^

## The global picture

The burden of disease attributable to mental disorders is increasingly being recognised as a global health concern.^13^ It has been noted in recent years that, despite the growing recognition of the importance of mental health, progress and development of services has remained slow in low- and middle-income countries (LMICs).^13^ Five challenges for global mental health identified in 2018^13^ were:

• integrating mental health services into the community setting
• improving accessibility to effective psychotropic medications
• training multidisciplinary mental health professionals
• providing community-based care and rehabilitation for people with chronic mental disorders
• strengthening the mental health competence of all health professionals.

The slow progress in developing mental healthcare services in LMICs^13^ has resulted in calls for continued evaluation and analysis of the barriers to improving mental healthcare in order to best inform future initiatives and policy.

## Conclusions

The five challenges^13^ listed above are of great relevance to developing mental healthcare services in Cambodia, and the importance of developing community care is recognised in the Mental Health and Substance Misuse Strategic Plan.^11^ The initial growth in the number of trained mental health professionals and services was an extraordinary achievement. Despite this, it is widely acknowledged that the treatment gap remains wide for people with mental disorders in Cambodia and the government's mental health plan is yet to be fully implemented.^2^ Training opportunities need to be restarted and maintained, together with efforts to prevent attrition of professionals. More resources are needed to address the treatment gap, improve the quality of mental health services and implement school- and community-based preventive initiatives.^13^
